# Knockdown of lncRNA LINC00662 suppresses malignant behaviour of osteosarcoma cells via competition with miR-30b-3p to regulate ELK1 expression

**DOI:** 10.1186/s13018-022-02964-2

**Published:** 2022-02-05

**Authors:** Bin Wang, Zhengfeng Xu, Xiuhui Wang, Shengli Xia, Pan Cai, Minghui Wang, Zhenchao Gao

**Affiliations:** 1 Department of Orthopedics, Shanghai University of Medicine and Health Sciences Affiliated Zhoupu Hospital, No. 1500, Zhouyuan Road, Pudong New District, Shanghai City, 201318 China; 2 Department of Orthopedics, Shanghai Public Health Clinical Center, No. 2901, Caolang Road, Jinshan District, Shanghai City, 201508 China

**Keywords:** Osteosarcoma, microRNAs, Long non-coding RNA LINC00662, miR-30b-3p, ELK1

## Abstract

**Purpose:**

Osteosarcoma is a type of bone malignancy that mainly occurred in teenagers. This investigation is aimed to clarify the effect of long non-coding RNA (lncRNA) LINC00662 on the proliferation, migration, and invasion in osteosarcoma and explore the underlying action mechanisms.

**Methods:**

The mRNA expression of LINC00662 was determined by real-time quantitative polymerase chain reaction. Cell proliferation, migration, and invasion were evaluated by 3-(4,5-Dimethylthiazol-2-yl)-2,5-diphenyltetrazolium bromide, wound healing, and transwell assays, respectively. A dual-luciferase reporter assay was used to validate the target relationships Between microRNA (miR)-30b-3p and LINC00662/ ETS domain-containing protein 1 (ELK1). Western blotting was performed to determine the protein expression of ELK1. Xenograft model was established to evaluate the effects of LINC00662 silencing on tumor growth in vivo.

**Results:**

LncRNA LINC00662 and ELK1 were significantly increased, while miR-30b-3p was reduced in osteosarcoma tissues. The results of functional experiments indicated that transfection of small hairpin (sh)-LINC00662 and miR-30b-3p mimics repressed the migration, invasion, and proliferation of osteosarcoma cells. LncRNA LINC00662 also appeared to sponge miR-30b-3p in order to affect the expression of ELK1. Simultaneously, there were weak negative correlations between the expression of miR-30b-3p and LINC00662/ELK1 in osteosarcoma tissues. Rescue experiments suggested that ELK1 overexpression and downregulation of miR-30b-3p reversed the suppressive effects of sh-LINC00662 on the cell migration, invasion, and proliferation in osteosarcoma.

**Conclusions:**

The current study indicated that knockdown of LINC00662 repressed cell migration, invasion, and proliferation through sponging miR-30b-3p to regulate the expression of ELK1 in osteosarcoma. These results may uncover a promising target for the treatment of osteosarcoma.

## Introduction

Osteosarcoma, which mainly involves the long tubular bone, is a malignant bone tumor that occurs primarily in adolescents and children [1]. It is generally characterized by destruction and high metastasis [2] with the global incidence of approximately 4.4 persons per million [3]. Although medical options such as radiation therapy, chemotherapy, and surgery have improved in recent decades [4], frequent recurrence and distant metastasis still impacted the therapeutic effect [5]. Additionally, traditional therapies have serious side effects in osteosarcoma, such as myelosuppression, auditory toxicity, leukopenia, and thrombocytopenia [6, 7]. Hence, there is an urgent need to explore the molecular mechanism referring to osteosarcoma progression and determine additional therapeutic targets for osteosarcoma.

The functions of long non-coding RNAs (lncRNAs) in the field of tumors have been extensively explored in recent years [8, 9]. LncRNAs are a subtype of RNAs longer than 200 nucleotides that lack protein-coding functions [10]. Increasing evidence has revealed that lncRNAs play crucial roles in regulating gene expression at different stages, including chromatin remodeling, transcription, and post-transcriptional regulation [11]. The important functions of small interfering RNAs in musculoskeletal homeostasis have been uncovered recently [12]. Additionally, numerous reports have shown that lncRNAs are involved in the progression of osteosarcoma [13]. For instance, silencing of lncRNA LINC00514 can repress the migration, invasion, proliferation of osteosarcoma cells [14]. The results of a knockdown experiment indicated that suppression of lncRNA GHET1 attenuates the cell proliferation, migration, and invasion abilities in osteosarcoma [15]. In addition, lncRNA LINC00662, located on human chromosome 19q11 [16], has been verified as an oncogene in many cancers, including gastric cancer [17], lung cancer [18] and prostate cancer [19]. Notably, a recent study conducted by Liu et al. indicated that LINC00662 knockdown attenuates the proliferation, migration, and invasion of osteosarcoma cells by regulating the microRNA (miR)-15a-5p/Notch2 axis [20]. Because the pathogenesis of osteosarcoma is complex, some other downstream targets of LINC00662 are needed to be explored.

MiRNAs, a class of non-coding RNAs with 18–23 nucleotides in length, can suppress the expression of target genes by restraining messenger RNA translation or regulating the degradation of mRNAs [21]. Notably, lncRNAs can exert their functions in many cancers through sponging miRNAs [22, 23]. For example, silencing of LINC00662 represses cell migration, invasion, and proliferation by regulating miR-34a in prostate cancer [19]. LINC00662 promotes tumorigenesis by sponging miR-497-5p in colorectal cancer [24]. Meanwhile, miRNAs are also confirmed to be deeply involved in musculoskeletal homeostasis and inflammation, such as miR-499, miR-608, miR-146a, and miR-210 [25, 26]. Notably, miR-30 family is an important member of miRNA family containing 6 mature miRNA molecules (miR-30a, miR-30b, miR-30c-1, miR-30c-2, miR-30d, and miR-30e). This miRNA family has been reported to exert crucial functions in musculoskeletal disorders [27]. Furthermore, an increasing number of studies have revealed that miR-30b-3p, a member of miR-30 family, is related to the progression of several types of human cancers [28, 29]. It has also been reported that overexpressed miR-30b-3p remarkably suppresses the cell proliferation, invasion, and migration abilities in hepatocellular carcinoma (HCC) [28] and glioma cells [30]. However, despite the fact that lncRNA LINC00662 has been confirmed to play crucial roles in many cancers, there is currently no evidence regarding the function of miR-30b-3p and the regulation of miR-30b-3p by LINC00662 in osteosarcoma [17, 18, 31].

ETS domain-containing protein 1 (ELK1), a member of the ternary complex factor (TCF) subfamily of the ETS oncogene family, regulates the oncogene c-fos by phosphorylation through activation of the PKC/ERK pathways [32–35]. ELK1 is known as a major risk factor gene in osteosarcoma progression. A study conducted by Su et al. reported that ELK1 can trigger the high expression of MIR100HG, which is a factor for the poor prognosis of osteosarcoma patients [36]. In addition, ELK1 has been found serve as an oncogene regulated by miRNAs to promote tumour progression in various human cancers, such as miR-326-ELK1 or miR-330-5p-ELK1 in cervical cancer [37, 38], miR-2682-5p-ELK1 in bladder cancer [39], and miR-597-5p-ELK1 in pancreatic cancer [40]. Notably, previous studies have been demonstrated that miR-503-5p or miR-134 can target ELK1 to modulated osteosarcoma progression and chemoresistance [41, 42]. However, whether ELK1 is regulated by LINC00662/miR-30b-3p axis to be involved in osteosarcoma and its underlying mechanism are still unknown.

In the present study, the expression levels of LINC00662, miR-30b-3p, and ELK1, as well as the effects of LINC00662 and miR-30b-3p on the malignant behaviour of osteosarcoma cells were determined. Furthermore, the interactions among LINC00662, miR-30b-3p, and ELK1 in osteosarcoma were also explored. This investigation is aimed to verify whether LINC00662 could suppress the progression of osteosarcoma by regulating the miR-30b-3p/ELK1 axis. Our findings may provide a novel target for osteosarcoma treatment.

## Methods

### Osteosarcoma specimens

In the present study, 56 patients with OS (age range, 6–51; 29 male patients and 27 female patients) were recruited from May 2017 and January 2019 in the Shanghai University of Medicine and Health Sciences Affiliated Zhoupu Hospital. The patients underwent resection and the specimens were collected. None of the patients received chemotherapy or radiotherapy treatment prior to surgery. Specimen collection was obtained with the written informed consent of the patients or their families. The collected osteosarcoma tissues and adjacent tissue controls (> 3 cm from the edge of the tumor) were immediately stored in liquid nitrogen for subsequent use. This study was conducted in accordance with the Declaration of Helsinki and approved by the ethics committee of our hospital (no. 2020-C-068-E01).

### Cell culture

Human normal osteoblast cell line hFOB and OS cell lines (U2OS, MG63, 143B, and HOS) were purchased from the American Type Culture Collection (Manassas, VA, USA), cultured in Dulbecco’s Modified Eagle’s Medium supplemented with 10% foetal bovine serum (FBS) and maintained in a humidified atmosphere containing 5% CO_2_ at 37 °C. The cultured cells showed monolayer growth, and the adherence rate of the cells was 90% during passage.

### Real-time quantitative polymerase chain reaction (RT-qPCR)

Total RNA was extracted using the miRNeasy Mini Kit (Qiagen, Hilden, Germany) according to the manufacturer’s protocol. To determine the concentration and purity of RNA, RNA samples (5 μL) were diluted with RNase-free ultrapure water 20 times in order to read the optical density (OD) values at 260 nm and 280 nm using an ultraviolet spectrophotometer. The OD260/OD280 ratio was between 1.7 and 2.1, indicating that the purity was high and could meet the needs of subsequent experimental studies. The complementary DNA (cDNA) template was synthesised using the cDNA Reverse Transcription Kit from Applied Biosystems (Foster City, CA, USA), and then RT-qPCR was conducted with the SYBR Green PCR kit (Takara, Dalian, China). The reaction conditions were as follows: 10 min at 95 °C, 40 cycles of 95 °C for 10 s, 60 °C for 20 s, and 72 °C for 34 s. All primers purchased from Invitrogen (Carlsbad, CA, USA) were shown in Table 1. β-actin and U6 were chosen as internal references. The relative expression levels of LINC00662, miR-30b-3p, and ELK1 were calculated using the 2^−ΔΔCt^ method.

**Table 1:** Primers for qRT-PCR

Gene	Forward	Reverse
LINC00662	5’-ACTAACAAGCTGGGTGCAGA-3’	5’-CCTCCTGGTCTGCGAGAAAT-3’
miR-30b-3p	5’-TGCGGAGAGGTTGCCCTTGGTGA-3’	5’-TGCGGGTGCTCGCTTCGGCAGC-3’
ELK1	5’-CCTTGCGGTACTACTATGAC-3’	5’-GGCTGCGGCTGCAGAGACTGG-3’
β-actin	5’-CTTAGTTGCGTTACACCCTTTCTTG-3’	5’-CTGTCACCTTCACCGTTCCAGTTT-3’

### Cell transfection

Small hairpin (sh)-LINC00662, the negative control vector (sh-NC), miR-NC, miR-30b-3p mimics, inhibitor NC, miR-30b-3p inhibitor, pcDNA-NC, and pcDNA-ELK1 were purchased from RiboBio Company (Beijing, China). When the cultured cells reached 80–90% confluence, the above factors were transfected into U2OS and MG63 cells using Lipofectamine 3000 Transfection Reagent (Invitrogen) for 48 h. The sequences of transfected fragment were as follows: miR-NC, 5’-CAGUACUUUUGUGUAGUACAA-3’; miR-30b-3p mimics, 5’-UGUAAACAUCCUACACUCAGCU-3’; inhibitor NC, 5’-UCACAACCUCCUAGAAAGAGUAGA-3’; miR-30b-3p inhibitor, 5’-AGCUGAGUGUAGGAUGUUUACA-3’; sh-LINC00662, 5’-GCUGCUGCCACUGUAAUAAUU-3’; sh-NC, 5’-AAUUCUCGGAACGUCUGACGU-3’.

### Target prediction

The miRNA targets of LINC00662 were predicted using LncBase Predicted v.2 software (http://carolina.imis.athena-innovation.gr/diana_tools/web/index.php?r=lncbasev2/index-predicted), and 2987 targets were predicted. Among these miRNA targets, miR-30b-3p was selected for the following assays because of its important role in human cancers and unknown regulatory relationship with LINC00662. In addition, the mRNA targets of miR-30b-3p were further predicted using miRDB software (http://mirdb.org/), and 1207 targets were predicted. ELK1 was selected for the following assays because of its important role in osteosarcoma.

### Dual-luciferase reporter (DLR) assay

The 3'-untranslated region (UTR) reporter constructs (wild-type [WT]) of LINC00662 and ELK1 (LINC00662-WT and ELK1-WT) harbouring the complementary sequence of miR-30b-3p as well as 3'-UTR reporter constructs (mutant type [MUT]) of LINC00662 and ELK1 (LINC00662-MUT and ELK1-MUT) containing the mutant sequence of miR-30b-3p were purchased from Hanbio (Shanghai, China). MG63 and U2OS cells were transfected with the reporter vectors, along with miR-30b-3p mimics or miR-NC. A DLR assay system (Promega, Madison, WI, USA) was used to measure the relative luciferase activity at 48 h after transfection.

### 3-(4,5-Dimethylthiazol-2-yl)-2,5-diphenyltetrazolium bromide (MTT) assay

MG63 and/or U2OS cells (5 × 10^3^ cells/well) were plated into 96-well plates. At 0, 24, 48, 72, and 96 h after cell culture, 20 μL MTT solution (5 mg/mL) was added to each well. Following incubation for 4 h, the medium was dumped, and 100 μL dimethyl sulfoxide was added to each well. A microplate reader (MG LABTECH, Durham, NC, USA) was used to measure the OD of cell lysates at 490 nm.

### Wound healing assay

MG63 and/or U2OS cells were seeded into 6-well plates (6 × 10^5^ cells/well) and cultured at 37 °C in an incubator with 5% CO_2_ until the cells reached 100% confluence. A sterile pipette tip (200 µL) was used to scrape the monolayer of cells to make a wound. The cells were continuously cultured in serum-free medium for 24 h. Finally, the cells were observed under an inverted microscope (TE2000; Nikon, Tokyo, Japan) and photographed at 0 and 24 h after wounding to measure the wound-healing distance. The formula for calculating the wound healing rate is as follows: (24 h scratch width/0 h scratch width) × 100.

### Transwell assay

The invasion ability of cells was measured using a transwell chamber (Corning, Corning, NY, USA) with an 8 µm pore size and Matrigel. First, 1 × 10^5^ cells in serum-free medium were placed in the upper chamber pre-coated with Matrigel, and the lower chamber was filled with 500 µL culture medium containing 20% FBS. After 24 h of culture at 37 °C with 5% CO_2_, 0.5% crystal violet solution (Sigma-Aldrich, St. Louis, MO, USA) was applied to stain the invasive cells on the lower surface of the insert membrane. After washing with PBS, the number of invasive cells was calculated in 5 randomly selected fields under an inverted microscope (Olympus, Tokyo, Japan).

### Western blot

Total proteins were extracted from the tissues and cells using radioimmunoprecipitation assay buffer (Beyotime, Shanghai, China). Equal amounts of proteins were separated by 10% sodium dodecyl sulphate polyacrylamide gel electrophoresis and transferred onto a polyvinylidene fluoride membrane (Merck Millipore, Billerica, MA, USA). The membranes were blocked with 5% skim milk. Then, the protein samples were incubated with the primary antibodies including anti-ELK1 (1:1000; LM16850; Santa Cruz Biotechnology, Beijing, China) and anti-β-actin (1:4000; ab115777; Abcam, Cambridge, MA, USA) overnight at 4 °C. After the membranes were washed with tris-buffered saline-Tween 20 three times, a secondary antibody (1:5000, ab6728; Abcam) was added and incubated with the protein samples at 37 °C for 2 h. The relative expression of ELK1 was normalized to that of the endogenous control β-actin using Image Lab™ software (Bio-Rad, Hercules, CA, USA).

### Xenograft model in vivo

BALB/c nude mice (male, 6–8 weeks, 21–25 g) were procured from EseBio (Shanghai, China). All the mice were housed in a controlled environment (21 ± 1 °C, 60% humidity, 12/12 h light/dark cycle), and had free access to food and water. Experimental procedures were conducted on the basis of the Institutional Animal Care and Use Committee of the Shanghai University of Medicine & Health Sciences Affiliated Zhoupu Hospital. Thereafter, the mice were divided into two groups ad libitum, including the sh-NC group (*n* = 5) and sh-LINC00662 (*n* = 5) group. MG63 cells (1 × 10^6^ cells/100 μL PBS) transfected with sh-NC or sh-LINC00662 were subcutaneously injected into each mouse. Then tumor size was recorded on the 7th, 14th, 21th and 28th day, and tumor volume was calculated according to the following formula: volume = width^2^ × length/^2^. On the 28th day after injection, mice were anesthetized by an intraperitoneal injection of 50 mg/kg pentobarbital sodium, and then sacrificed by cervical dislocation. The tumors were collected and weighed.

### Immunohistochemistry (IHC) assay

IHC staining was conducted using streptavidin–biotin-peroxidase complex method. Briefly, osteosarcoma samples were fixed, paraffin-embedded, dewaxed, rehydrated, and antigen retrieval. Then samples were stained with primary antibody Ki67 (1: 1500; Abcam, Cambridge, UK) at 4 °C overnight, followed by incubation with HRP-conjugated secondary antibody (1:3000; Abcam) for 30 min at 37 °C. The Ki67-positive cells exhibited brown punctate granules in the nucleus. Pictures were taken under a light microscope (magnifications, × 200).

### Statistical analysis

**Fig. 1:**
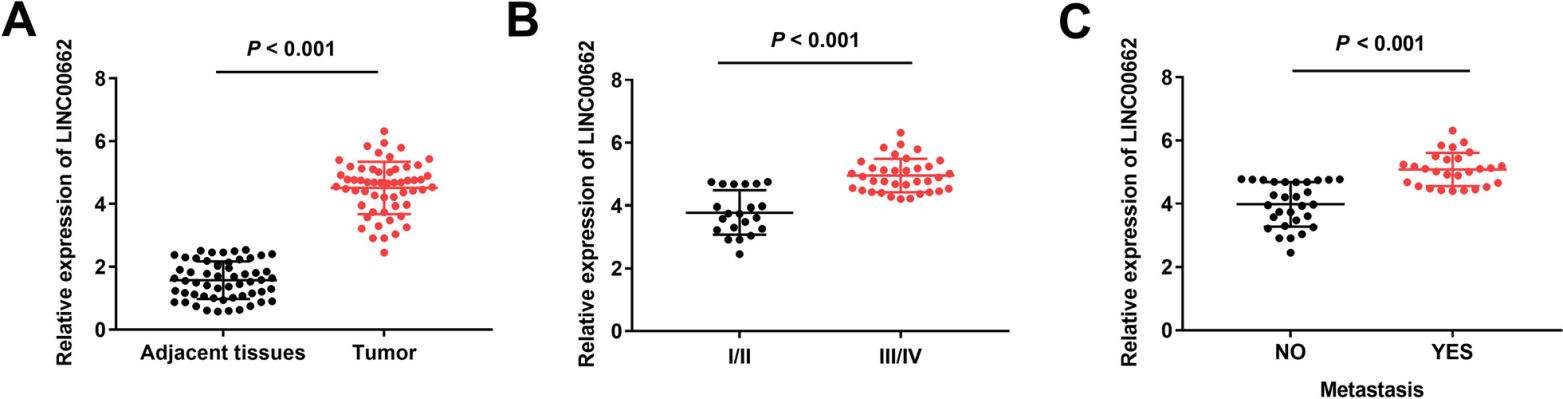
The expression of lncRNA LINC00662 is elevated in osteosarcoma tissues. **A** Relative expression of LINC00662 was detected by qRT-PCR in tumor tissues and adjacent normal tissues. *P* < 0.001, versus adjacent normal tissues. **B** Relative expression of LINC00662 in tumor tissues with WHO grade I/II and WHO grade III/IV was determined by qRT-PCR. *P* < 0.001, versus I/II. **C** Relative expression of LINC00662 in tumor tissues with metastasis and without metastasis was determined by qRT-PCR. *P* < 0.001, versus NO

**Fig. 2:**
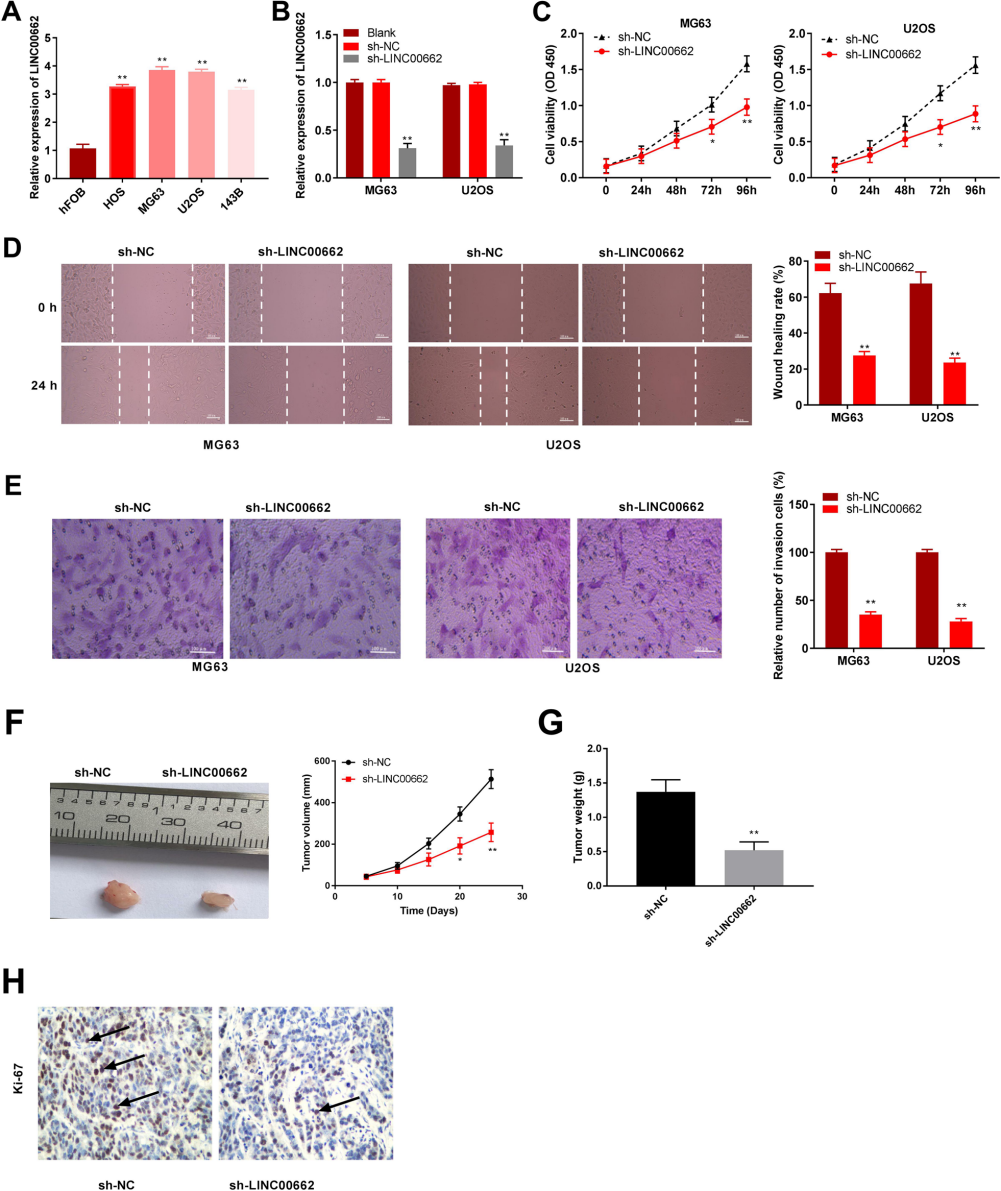
Knockdown of lncRNA LINC00662 represses proliferation, migration and invasion of osteosarcoma cells. **A** Relative expression of LINC00662 in osteosarcoma cell lines was detected by qRT-PCR. **P < 0.01, versus hFOB. **B** Relative expression of LINC00662 in U2OS and MG63 cell lines after transfection with sh-LINC00662 was detected by qRT-PCR. **P < 0.01, versus sh-NC. **C** The cell viability in U2OS and MG63 cell lines was detected by MTT assay. *P < 0.05, **P < 0.01, versus sh-NC. **D** Wound healing rate was detected by wound healing assay. **P < 0.01, versus sh-NC. Scale bar = 100 µm. **E** Relative number of invasion cells was detected by transwell assay. **P < 0.01, versus sh-NC. Scale bar = 100 µm. **F** The solid tumor image and tumor volume in xenograft mice after injection of sh-NC or sh-LINC00662. *P < 0.05, **P < 0.01, versus sh-NC. **G** Tumor weight in xenograft mice after injection of sh-NC or sh-LINC00662. **P < 0.01, versus sh-NC. **H** Immunohistochemistry (IHC) staining of Ki-67 in xenograft mice generated by sh-NC or sh-LINC00662 injection. magnifications, × 200

**Fig. 3:**
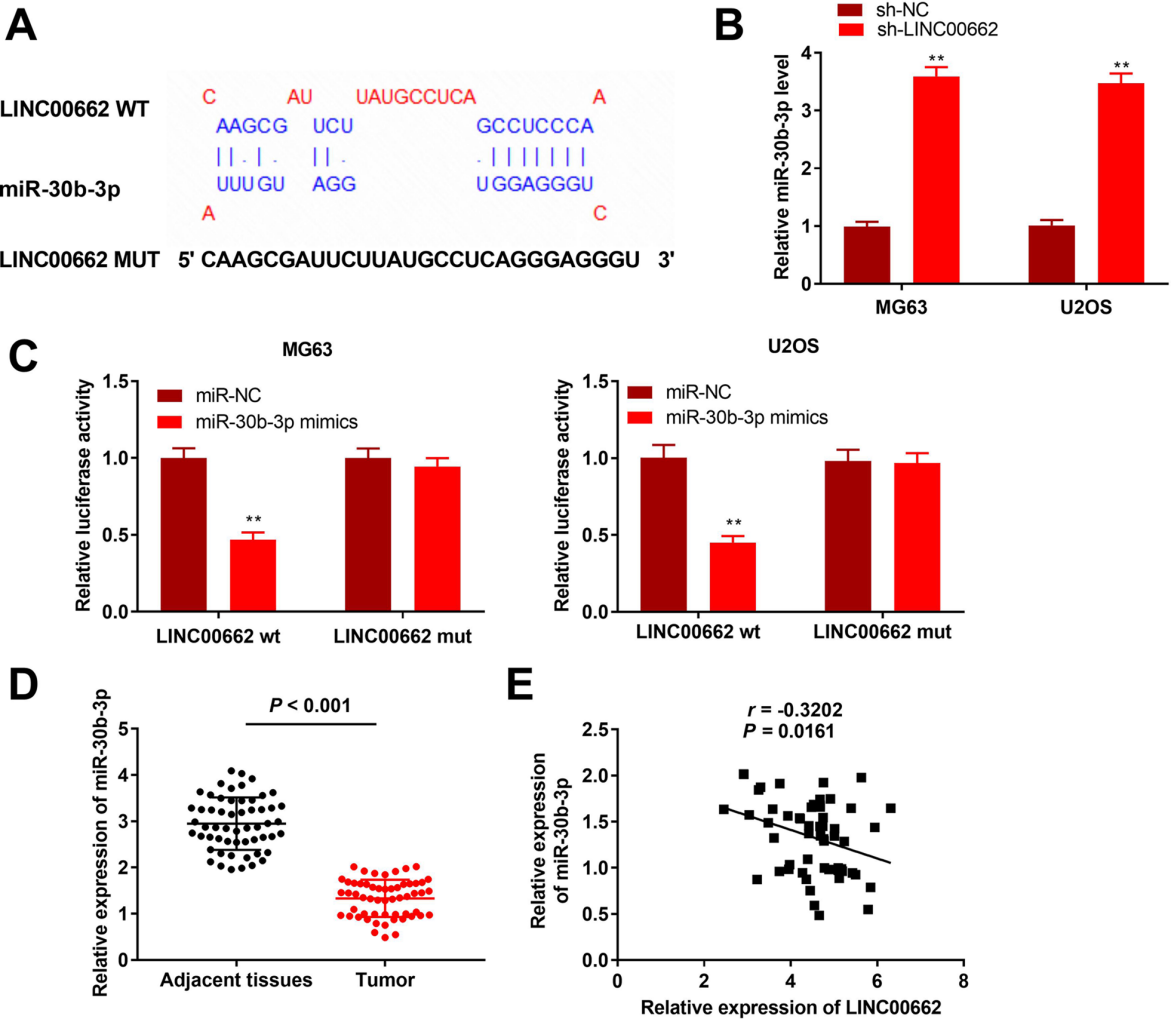
MiR-30b-3p is targeted by LINC00662. **A** The binding sequence between LINC00662 and miR-30b-3p was predicted by starbase2.0. **B** Relative miR-30b-3p level was detected by qRT-PCR. **P < 0.01, versus sh-NC. **C** Relative luciferase activity of LINC00662 vector was detected by DLR assay. **P < 0.01, versus miR-NC. **D** Relative expression of miR-30b-3p in tumor tissues and adjacent normal tissues was detected by qRT-PCR. *P* < 0.001, versus adjacent normal tissues. **E** Correlation analysis of LINC00662 and miR-30b-3p in osteosarcoma tissues. *P* = 0.0161, r =  − 0.3202

**Fig. 4:**
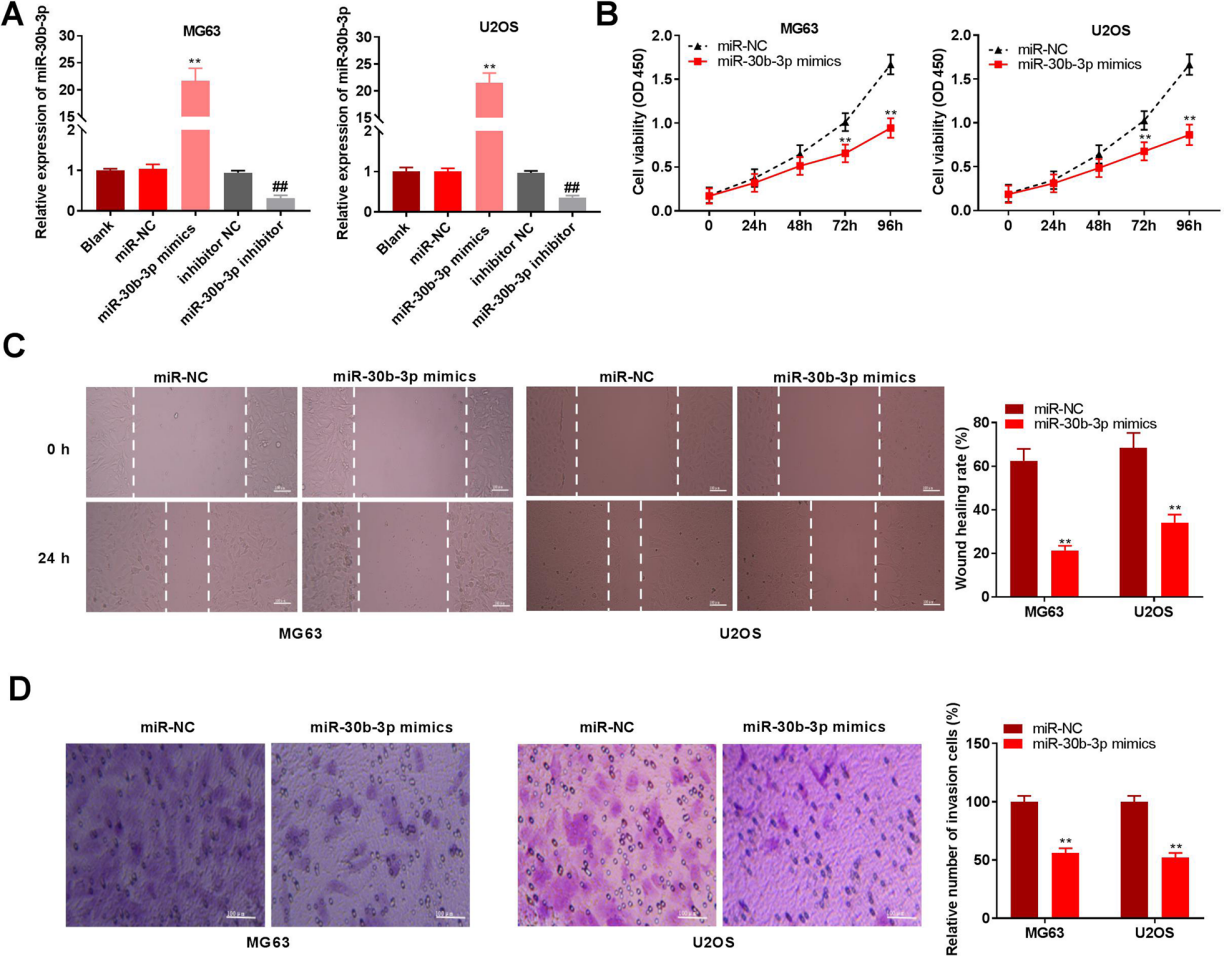
MiR-30b-3p inhibits proliferation, migration and invasion of osteosarcoma cells. **A** Relative expression of miR-30b-3p was detected by qRT-PCR. **P < 0.01, versus miR-NC; ## *P* < 0.01, versus inhibitor NC. **B** The cell viability was determined by MTT in U2OS and MG63 cells. **P < 0.01, versus miR-NC. **C** Wound healing rate was determined by wound healing rate. **P < 0.01, versus miR-NC. Scale bar = 100 µm. **D** Relative number of invasion cells was detected by transwell assay. **P < 0.01, versus miR-NC. Scale bar = 100 µm

**Fig. 5:**
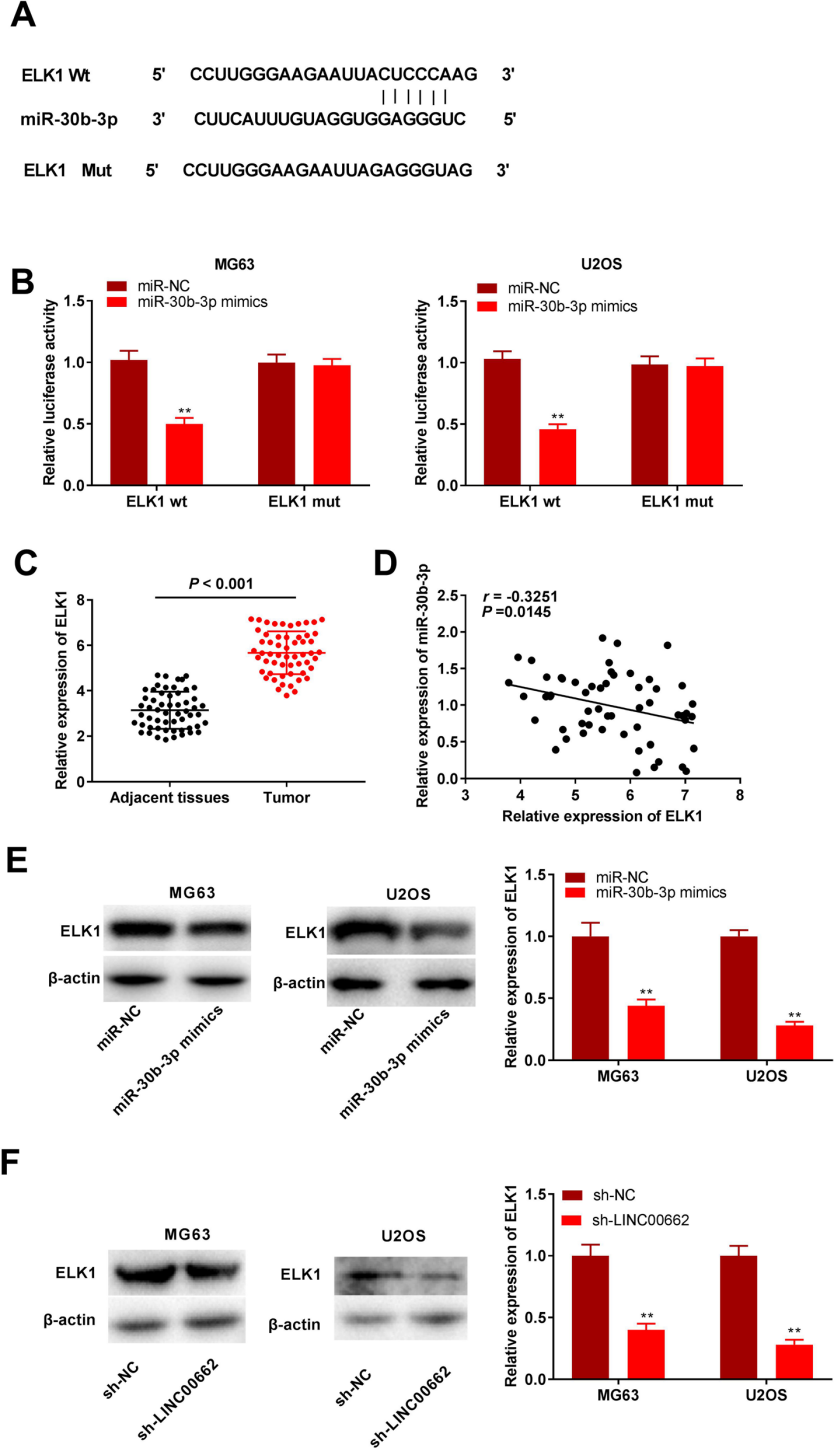
MiR-30b-3p can target ELK1. **A** The binding sites between miR-30b-3p and ELK1 were predicted by starbase2.0. **B** Relative luciferase activity of ELK1 vector was detected by DLR assay. **P < 0.01, versus miR-NC. **C** qRT-PCR was used to detect relative expression of ELK1 in osteosarcoma tissues and adjacent normal tissues. *P* < 0.001, versus adjacent normal tissues. **D** Correlation of ELK1 and miR-30b-3p expression in osteosarcoma tissues was analyzed by Pearson's correlation analysis. *P* = 0.0279, *r* =  − 0.2939. **E** After transfection with miR-30b-3p mimics or miR-NC in MG63 and U2OS cells, expression of ELK1 was detected by western blot. **P < 0.01, versus miR-NC. **F** After transfection with sh-LINC00662 or sh-NC in MG63 and U2OS cells, protein level of ELK1 was measured by western blot. **P < 0.01, versus sh-NC

All experiments were independently repeated three times. SPSS Statistics 22.0 software (IBM SPSS, Armonk, NY, USA) was utilized for the statistical analysis. All data are presented as mean ± standard deviations. A one-way analysis of variance was used for the comparisons among multiple groups, and Tukey’s multiple comparisons test was used for pairwise comparisons. Comparisons between two groups were performed using Student’s *t*-test (paired: Figs. 1A, 3D and 5C; unpaired: Figs. 1B, C, 2D, E, 3B, 4C, D and 5E). Pearson correlation analysis was used to assess the correlation between miR-30b-3p and LINC00662/ELK1. The level of significance was set at *P* < 0.05.

## Results

### LncRNA LINC00662 is highly expressed in osteosarcoma samples

First, the expression of lncRNA LINC00662 in osteosarcoma samples was evaluated by RT-qPCR, which indicated that LINC00662 was markedly upregulated in tumor tissues compared to adjacent normal tissues (*P* < 0.001; Fig. 1A). In addition, we discovered that lncRNA LINC00662 was closely related to World Health Organization (WHO) tumor grade and metastasis (*P* < 0.05; Table 2). LINC00662 was increased in tumor tissues with WHO grade III + IV compared to those with WHO grade I + II (*P* < 0.001; Fig. 1B). LINC00662 was also elevated in tumor tissues with metastasis relative to those without metastasis (*P* < 0.001; Fig. 1C).

**Table 2:** Correlations between clinicopathological features and the expression of LINC00662 in OS tissues

Characteristics	Total	LINC00662 expression		*P*-value
		Low (28)	High (28)	
*Age*				0.592
< 18 years	30	14	16	
≥ 18 years	26	14	12	
*Gender*				0.789
Male	29	15	14	
Females	27	13	14	
*Tumor size*				0.284
< 8 cm	26	15	11	
≥ 8 cm	30	13	17	
*Histological subtype*				0.849
Osteoblastic	21	11	10	
Chondroblastic	17	9	8	
Fibroblastic	18	8	10	
*Metastasis*				0.016*
No	29	19	10	
Yes	27	9	18	
*WHO grade*				0.013*
I + II	21	15	6	
III + IV	35	13	22	

*P < 0.05, WHO: World Health Organization

### Knockdown of lncRNA LINC00662 inhibits the migration, invasion, and cell growth in osteosarcoma

The RT-qPCR results demonstrated that high expression of LINC00662 was presented in the HOS, 143B, U2OS, and MG63 cell lines compared to the hFOB cell line (*P* < 0.01; Fig. 2A). Next, sh-LINC00662 or sh-NC was transfected into MG63 and U2OS cells, and the transfection efficiency of sh-LINC00662 was detected by RT-qPCR. We found that the relative expression of LINC00662 was significantly reduced in U2OS and MG63 cells after transfection (*P* < 0.01; Fig. 2B). To investigate the role of LINC00662 in osteosarcoma cells, MTT, wound healing, and transwell assays were performed. The MTT assay results revealed that cell viability decreased at 96 h after transfection of sh-LINC00662 into U2OS and MG63 cells (*P* < 0.01; Fig. 2C). Meanwhile, the wound healing rate and relative number of invasion cells were remarkably reduced by transfection of sh-LINC00662 into U2OS and MG63 cells (*P* < 0.01; Fig. 2D, E). Additionally, the effect of LINC00662 silencing on tumor xenograft was further explored. As illustrated in Fig. 2F, the tumor volume was smaller in the sh-LINC00662 group compared to the sh-NC group (*P* < 0.05). Meanwhile, tumor weight was also reduced in the sh-LINC00662 group compared to that of the sh-NC group (*P* < 0.01; Fig. 2G). Additionally, IHC analysis showed that Ki67 staining was remarkably alleviated by injection of sh-LINC00662 (Fig. 2H).

### LINC00662 acts as a sponge of miR-30b-3p

To elucidate the molecular mechanism by which sh-LINC00662 suppressed the cell behaviour of osteosarcoma, LncBase Predicted v.2 software was applied to explore the miRNAs that interact with LINC00662. We demonstrated that LINC00662 harboured a target sequence of miR-30b-3p (Fig. 3A). The RT-qPCR data revealed that miR-30b-3p was markedly elevated by LINC00662 knockdown in the U2OS and MG63 cell lines (*P* < 0.01; Fig. 3B). To validate the relationship between LINC00662 and miR-30b-3p, a DLR assay was performed. We discovered that miR-30b-3p overexpression notably repressed the relative luciferase activity of LINC00662-WT while the site-directed MUT of the miR-30b-3p binding sequence markedly abolished the effect of miR-30b-3p on the expression of the reporter gene in the U2OS and MG63 cell lines (*P* < 0.01; Fig. 3C). MiR-30b-3p was found to be downregulated in tumor tissues compared to the adjacent normal tissues (*P* < 0.001; Fig. 3D). Furthermore, we discovered that there was a weak negative correlation between the expression of miR-30b-3p and LINC00662 in osteosarcoma tissues (*P* = 0.0161 and *r* =  − 0.3202; Fig. 3E). The above outcomes established the assumption that LINC00662 serves as a molecular sponge of miR-30b-3p in osteosarcoma.

### MiR-30b-3p represses the abilities to proliferate, migrate, and invade in osteosarcoma cells

The RT-qPCR results showed that miR-30b-3p expression was increased by miR-30b-3p mimics transfection, whereas was decreased after transfection of miR-30b-3p inhibitor in U2OS and MG63 cell lines (*P* < 0.01; Fig. 4A). Overexpression of miR-30b-3p decreased cell viability in osteosarcoma cells (*P* < 0.01; Fig. 4B). The migratory and invasive abilities of osteosarcoma cells were also remarkably inhibited by overexpression of miR-30b-3p (*P* < 0.01; Fig. 4C, D). The above data suggest that miR-30b-3p may exert the role of a tumour suppressor in osteosarcoma cells.

### miR-30b-3p targets ELK1

The miRDB database showed that the 3′-UTR of ELK1 had binding sites to bind miR-30b-3p (Fig. 5A). The DLR assay revealed that overexpression of miR-30b-3p reduced the luciferase activity of the WT vector ELK1, and the luciferase activity of the MUT vector of ELK1 was not markedly different, indicating that ELK1 was a downstream target of miR-30b-3p (*P* < 0.01; Fig. 5B). In addition, we found that ELK1 expression was remarkably elevated in tumor tissues compared to adjacent normal tissues (*P* < 0.001; Fig. 5C). Pearson’s correlation analysis indicated that miR-30b-3p was weakly negatively correlated with ELK1 (*P* = 0.0279 and r = − 0.2939; Fig. 5D). Western blot analysis revealed that ELK1 protein expression was reduced by overexpression of miR-30b-3p (*P* < 0.01; Fig. 5E) and downregulation of LINC00662 (*P* < 0.01; Fig. 5F). Taken together, our findings suggest that miR-30b-3p target and negatively modulate ELK1 expression.

### LINC00662 regulates cell invasion, migration, and proliferation via regulating the miR-30b-3p/ELK1 axis in osteosarcoma

**Fig. 6:**
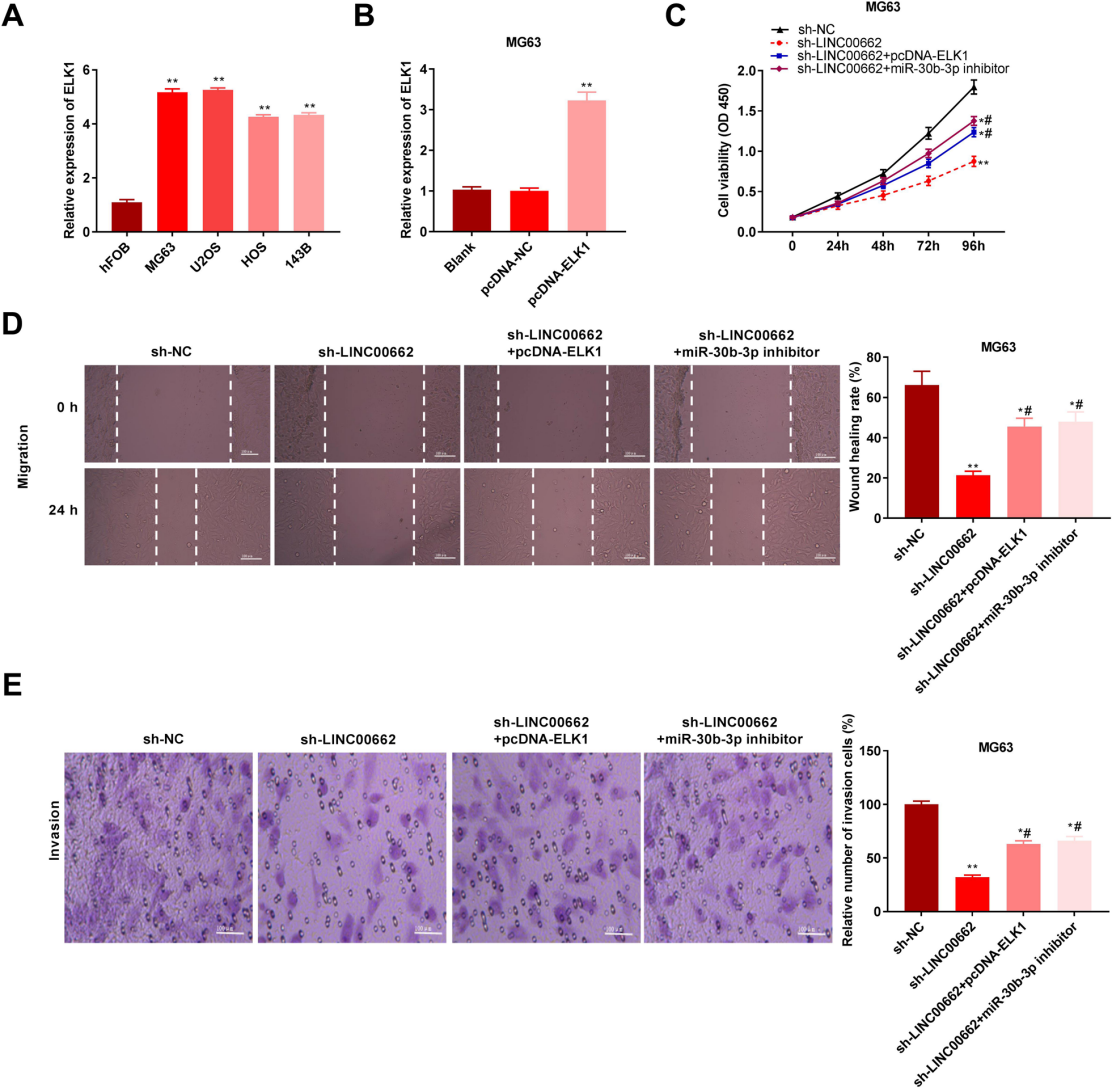
LncRNA LINC00662 regulates cell proliferation, migration and invasion via mediating miR-30b-3p/ELK1 axis. **A** Relative expression of ELK1 was detected by qRT-PCR. **P < 0.01, versus hFOB. **B** Relative expression of ELK1 was detected by qRT-PCR after transfection with pcDNA-ELK1. **P < 0.01, versus pcDNA-NC. **C** The cell viability was determined by MTT assay in MG63 cells. *P < 0.05, **P < 0.01, versus sh-NC. ^#^*P* < 0.05, versus sh-LINC00662. **D** Wound healing rate of cells was determined by wound healing assay in MG63 cells. *P < 0.05, **P < 0.01, versus sh-NC. ^#^*P* < 0.05, versus sh-LINC00662. Scale bar = 100 µm. **E** Relative number of invasion cells in MG63 cells was detected by transwell assay. *P < 0.05, **P < 0.01, versus sh-NC. ^#^*P* < 0.05, versus sh-LINC00662. Scale bar = 100 µm

ELK1 was highly expressed in the U2OS, MG63, 143B, and HOS cell lines compared to the hFOB cell line (*P* < 0.01; Fig. 6A). Transfection of pcDNA-ELK1 significantly promoted the expression of ELK1 in MG63 cells (*P* < 0.01; Fig. 6B). To verify the relationship between LINC00662 and the miR-30b-3p/ELK1 axis in osteosarcoma, rescue experiments were performed. We demonstrated that the inhibitory effects of sh-LINC00662 on the proliferative, migratory, and invasive capacities of MG63 cells were reversed by downregulation of miR-30b-3p and upregulation of ELK1 (*P* < 0.05; Fig. 6C–E).

## Discussion

Upregulation of several lncRNAs has been found to be involved in osteosarcoma progression [43, 44]. Prior studies have confirmed that lncRNAs such as LINC00689 [45], SNHG1 [46] and HOST2 [47] are markedly overexpressed in osteosarcoma tissues and cells. Similarly, lncRNA LINC00662 was also upregulated in osteosarcoma tissues and cells, which is consistent with the results of previous studies. Additionally, Li et al. stated that LINC00662 is positively associated with distant metastasis in prostate cancer [19]. In this study, we observed that high expression of LINC00662 was not only associated with metastasis but also correlated with WHO grade in osteosarcoma patients. These results imply that LINC00662 may be a pathogenic factor in the tumorigenesis of osteosarcoma. However, the sample sizes in our study were relatively small, and further research on additional patients with osteosarcoma is required to verify our outcomes.

Recently, functional assays of LINC00662 in many cancers have been performed, and the results have indicated that LINC00662 knockdown acts as a tumor suppressor [19, 48]. For instance, knockdown of LINC00662 suppresses cell growth, migration, and invasion by mediating the epithelial-mesenchymal transition pathway in colorectal cancer [24] and oral squamous cell carcinoma [31]. Here, we found that sh-LINC00662 repressed the abilities of osteosarcoma cells to proliferate, migrate, and invade in vitro and inhibited the growth of tumor xenograft in vivo. In addition, Liu et al. demonstrated that LINC00662 downregulation attenuates osteosarcoma progression by sponging miR-15a-5p [20]. In this study, we further determined miR-30b-3p is a target of LINC00662. Therefore, we speculate that miR-30b-3p may also be regulated by LINC00662 in order to mediate the occurrence of osteosarcoma.

MiR-30b-3p belongs to the miR-30 family [49] and results from various studies have shown that other miR-30 family members (miR-30a and miR-30c) are downregulated in osteosarcoma tissues [50, 51]. In the current study, we discovered that miR-30b-3p expression was decreased in osteosarcoma tissues. In addition, existing studies have shown that overexpressed miR-30b-3p represses the abilities of cells to proliferate, migrate and invade in ovarian cancer [52] and hepatocellular carcinoma [28]. Here, our findings also showed that overexpressed miR-30b-3p repressed cell proliferation, invasion, and migration in osteosarcoma. In addition, we found that there was a weak inverse correlation between miR-30b-3p and LINC00662, and the miR-30b-3p inhibitor reversed the suppressive effects of sh-LINC00662 on the cell proliferation, migration, and invasion abilities in osteosarcoma. In summary, we speculate that miR-30b-3p also interacts with LINC00662 to ameliorate the malignant behaviors of osteosarcoma.

ELK1 is a member of the ternary complex factor subfamily [53]. A previous study demonstrated that ELK1-induced upregulation of MIR100HG promotes the progression of osteosarcoma. In this study, we revealed that ELK1 was significantly increased in osteosarcoma tissues and cells, but the function of ELK1 was not explored. A growing number of studies have confirmed that ELK1 is the downstream target of miRNAs, including miRNA-135a [54], miRNA-873 [55] and miR-185-5p [56], in diverse cancers. In the present study, we identified ELK1 is a target of miR-30b-3p in osteosarcoma. Moreover, the results of Pearson’s correlation analysis showed that there was a weak negative correlation between miR-30b-3p and ELK1 in osteosarcoma tissues. Combined with the above outcomes, we determined that overexpressed miR-30b-3p exerted its effect as a tumor suppressor by targeting ELK1 in osteosarcoma. Meanwhile, we found that the suppressive effects of LINC00662 on the proliferation, migration, and invasion abilities of osteosarcoma cells were reversed by overexpression of ELK1. We inferred that sh-LINC00662 may exert anti-tumour role by mediating the miR-30b-3p/ELK1 axis in osteosarcoma. Additionally, ELK1 has been reported to interact with many signalling pathways in human cancers, such as the ELK1-CHOP/death receptor 5 pathway in colorectal cancer [57] and the ELK1-mitogen-activated protein kinase kinase (MEK)/extracellular signal-regulated kinase (ERK) pathway in hepatocellular carcinoma [58] or glioblastoma [59]. Notably, ELK1 can also interact with the p53/reactive oxygen species/ERK1/ERK2 [53] and Raf-1/MEK/ERK/twist-related protein 1 signalling pathways [60] in osteosarcoma. We speculated that the LINC00662/miR-30b-3p/ELK1 axis may be involved in the progression of osteosarcoma by regulating these signalling pathways. In addition, some potential downstream genes of miR-30b-3p, such as ATG5 [61], PAK1 [62], FOXP4 [63], and MYO10 [64] also exerted crucial roles in osteosarcoma. We speculated that these genes may be also involved in the regulatory mechanism of LINC00662/miR-30b-3p in osteosarcoma.

However, there were also some limitations in this study. First, the correlations between the LINC00662/miR-30b-3p/ELK1 axis and clinicopathological feature or survival of patients are not fully revealed. Second, researches on the regulatory mechanisms of LINC00662/miR-30b-3p axis involving more downstream genes and signalling pathways in osteosarcoma are needed. Third, this study only focuses on the cellular level, and further in vivo experiments should be performed. We will elucidate these issues in future studies.

In conclusion, lncRNA LINC00662 expression was strikingly enhanced in osteosarcoma tissues and cell lines and was correlated with WHO tumor grade and metastasis. Furthermore, knockdown of LINC00662 and overexpression of miR-30b-3p promoted cell proliferation, invasion, and migration in osteosarcoma. Bioinformatics methods and a DLR assay confirmed that LINC00662 could function as a sponge for miR-30b-3p, and ELK1 was the downstream target of miR-30b-3p in osteosarcoma. Our findings indicate that LINC00662 mediates the progression of osteosarcoma via competition with miR-30b-3p to regulate the expression of ELK1. Therefore, the study findings improved our understanding of the effect of LINC00662 on osteosarcoma and may provide a potential target for osteosarcoma treatment.

## Data Availability

All data are available through corresponding author Zhenchao Gao.
